# Integrated metabolic and microbial analysis reveals host–microbial interactions in IgE-mediated childhood asthma

**DOI:** 10.1038/s41598-021-02925-5

**Published:** 2021-12-03

**Authors:** Chih-Yung Chiu, Mei-Ling Cheng, Meng-Han Chiang, Chia-Jung Wang, Ming-Han Tsai, Gigin Lin

**Affiliations:** 1 Division of Pediatric Pulmonology, College of Medicine, Chang Gung Memorial Hospital at Linkou, Chang Gung University, Taoyuan, Taiwan; 2 Clinical Metabolomics Core Laboratory, Chang Gung Memorial Hospital at Linkou, Taoyuan, Taiwan; 3 Department of Biomedical Sciences, and Metabolomics Core Laboratory, Healthy Aging Research Center, College of Medicine, Chang Gung University, Taoyuan, Taiwan; 4 Department of Medical Imaging and Intervention, Imaging Core Laboratory, Institute for Radiological Research, and Clinical Metabolomics Core Laboratory, College of Medicine, Chang Gung Memorial Hospital at Linkou, Chang Gung University, Taoyuan, Taiwan; 5 Department of Pediatrics, College of Medicine, Chang Gung Memorial Hospital at Keelung, Chang Gung University, Taoyuan, Taiwan

**Keywords:** Paediatric research, Asthma, Metabolic syndrome, Molecular medicine

## Abstract

A metabolomics-based approach to address the molecular mechanism of childhood asthma with immunoglobulin E (IgE) or allergen sensitization related to microbiome in the airways remains lacking. Fifty-three children with lowly sensitized non-atopic asthma (n = 15), highly sensitized atopic asthma (n = 13), and healthy controls (n = 25) were enrolled. Blood metabolomic analysis with ^1^H-nuclear magnetic resonance (NMR) spectroscopy and airway microbiome composition analysis by bacterial 16S rRNA sequencing were performed. An integrative analysis of their associations with allergen-specific IgE levels for lowly and highly sensitized asthma was also assessed. Four metabolites including tyrosine, isovalerate, glycine, and histidine were uniquely associated with lowly sensitized asthma, whereas one metabolite, acetic acid, was strongly associated with highly sensitized asthma. Metabolites associated with highly sensitized asthma (valine, isobutyric acid, and acetic acid) and lowly sensitized asthma (isovalerate, tyrosine, and histidine) were strongly correlated each other (*P* < 0.01). Highly sensitized asthma associated metabolites were mainly enriched in pyruvate and acetyl-CoA metabolisms. Metabolites associated with highly sensitized atopic asthma were mostly correlated with microbiota in the airways. Acetic acid, a short-chain fatty acid (SCFA), was negatively correlated with the genus *Atopobium* (*P* < 0.01), but positively correlated with the genus *Fusobacterium* (*P* < 0.05). In conclusion, metabolomics reveals microbes-related metabolic pathways associated with IgE responses to house dust mite allergens in childhood asthma. A strong correlation of metabolites related to highly sensitized atopic asthma with airway microbiota provides linkages between the host–microbial interactions and asthma endotypes.

## Introduction

Asthma is a heterogeneous disease with a diverse genetic and environmental interaction^1^. Immunoglobulin E (IgE) production has demonstrated to be associated with allergen exposure and asthma, however, allergic symptom could be presented without elevation of serum IgE levels. Although there are more similarities than differences between atopic and non-atopic asthma, few studies have addressed their molecular characterization, especially in early childhood. Metabolic phenotyping provides a scientific research strategy to understand the interactions between the allergen exposure to IgE antibodies and asthma at the molecular level^2^. A metabolomics-based approach could contribute to the possibility of discovering biomarkers and molecular mechanism for different asthma endotypes.

Allergen exposure plays a significant role in the development of IgE sensitization and triggering of symptomatic allergies^3^. The human microbiota mediates the mucosal immune responses modulating the allergic response to allergens, which may differ in molecular immune reactions with different phenotypes of allergies^4^. Microbial dysbiosis in airways has reported to be associated with house dust mites (HDM), potentially contributing to the susceptibility to allergic asthma^5^. Several studies have identified altered host metabolic pathways in association with asthma^6^. However, the combined signature of the metabolome in responses to IgE sensitization and microbiome in airways for children with asthma has not yet been fully approached.

The host–microbe metabolic interactions are increasingly being associated with the development and manifestations of allergic diseases^7^. The aim of this study was to determine the plasma metabolic profiles using ^1^H-NMR spectroscopy in children with asthma and healthy controls. The relationship of metabolites involved in mediating allergen-induced IgE responses and asthma were assessed. The existence of the interactions of microbiota in the airways with the host metabolic processes and its implications in childhood asthma were also examined.

## Results

### Population characteristics

A total of fifty-three subjects were enrolled into this study, including 28 children with asthma (15 non-atopic, lowly sensitized asthma and 13 atopic, highly sensitized asthma), and 25 healthy controls. Table 1 shows the comparisons of baseline characteristics of children with asthma and healthy controls. There were statistically significant differences in total serum and mite-specific IgE levels among children with lowly sensitized asthma, highly sensitized asthma and healthy controls (*P* < 0.01).

**Table 1:** Comparison of the clinical and epidemiologic characteristics between lowly and highly sensitized asthma, and healthy controls.

Characteristics	Asthma		*P*-value	Asthma	Controls	*P*-value
	Non-atopic, lowly sensitized (n = 15)	Atopic, highly sensitized (n = 13)		Total (n = 28)	Healthy (n = 25)	
Age (yr)	3.7 ± 0.6	3.5 ± 0.7	0.374	3.6 ± 0.7	3.6 ± 0.7	0.887
Sex, male	9 (60.0%)	10 (76.9%)	0.435	19 (67.9%)	15 (60.0%)	0.552
BMI-for-age	16.0 ± 1.5	16.8 ± 1.6	0.199	16.3 ± 1.6	15.9 ± 2.1	0.519
Maternal atopy	5 (33.3%)	8 (61.5%)	0.136	13 (46.4%)	8 (32.0%)	0.284
Passive smoking	6 (40.0%)	9 (69.2%)	0.122	15 (53.6%)	9 (36.0%)	0.200
Breastfeeding > 6 mo*			0.755			0.074
Exclusive	5 (33.3%)	3 (23.1%)		8 (28.6%)	9 (36.0%)	
Partial	5 (33.3%)	4 (30.8%)		9 (32.1%)	13 (52.0%)	
Formula	5 (33.3%)	6 (46.2%)		11 (39.3%)	3 (12.0%)	
**Allergen-specific IgE, kU/L**						
*D. pteronyssinus*	2.0 ± 1.2	42.0 ± 37.4	** < 0.001**	20.6 ± 32.3	0.4 ± 0.3	**0.001**
*D. farinae*	1.0 ± 1.8	29.5 ± 36.1	** < 0.001**	14.2 ± 28.1	0.2 ± 0.3	**0.001**
Total serum IgE, kU/L	60.5 ± 71.4	401.3 ± 345.9	** < 0.001**	218.7 ± 292.9	38.3 ± 37.1	**0.001**

Data shown are mean ± SD or number (%) of patients as appropriate.

*yr* year, *BMI* body mass index, *IgE* immunoglobulin E.

Significance values are given in bold.

*Breastfeeding for the first 6 months of life.

### Identification of blood metabolites for lowly and highly sensitized asthma

A total of 64 buckets refer to 46 known metabolites were identified using Chenomx NMR Suite 8.0 professional software (Chenomx Inc., Edmonton AB, Canada). PLS-DA was used to identify metabolites discriminated between groups. Metabolites selected by using a FDR adjusted *P*-value < 0.05 in the fold change of expression level are shown in Table 2. Venn diagram showed the distribution of the metabolites in each comparison (Fig. S1). Among them, tyrosine, isovalerate, glycine, and histidine were associated with lowly sensitized non-atopic asthma, whereas acetic acid was uniquely associated with highly sensitized atopic asthma. Eight metabolites including lysine, citric acid, valine, glutamine, fumaric acid, isobutyric acid, pyruvic acid, and ethanol were associated with IgE levels related to asthma.

**Table 2:** The VIP score and fold change of metabolites significantly differentially expressed between children with lowly or highly sensitized asthma and controls, and between highly and lowly sensitized asthmatic children.

Metabolites	Chemical shift, ppm	Lowly sensitized asthma vs. controls			Highly sensitized asthma vs. controls			Highly vs. lowly sensitized asthma		
		VIP score*	Fold change^†^	*P*^‡^	VIP score	Fold change	*P*	VIP score	Fold change	*P*
Lysine	1.490–1.526(m)	1.70	0.64	**0.010**	0.18	1.09	0.952	1.45	1.71	**0.033**
Isovalerate	0.910–0.936(d)	1.57	0.75	**0.022**	0.08	1.03	0.976	1.35	1.38	0.058
Histidine	7.03–7.078(s)	0.96	1.15	**0.030**	0.32	1.11	0.060	0.30	0.96	0.683
Tyrosine	6.860–6.917(m)	1.01	0.87	**0.040**	0.06	0.98	0.952	0.63	1.13	0.052
Glycine	3.548–3.565(s)	2.78	1.16	**0.046**	0.70	1.09	0.411	0.19	0.94	0.496
Citric acid	2.494–2.555(d)	1.27	1.11	**0.049**	0.14	0.97	0.627	0.93	0.88	**0.046**
Ethanol	1.148–1.181(t)	0.64	1.35	0.804	0.77	2.01	**0.008**	0.41	1.49	**0.046**
Acetic acid	1.850–1.930(s)	0.69	1.33	0.956	0.50	1.50	**0.016**	0.21	1.12	0.201
Pyruvic acid	2.354–2.376(s)	1.00	1.13	0.562	1.14	0.68	**0.030**	0.68	0.60	**0.008**
Isobutyric acid	1.050–1.077(d)	0.65	0.74	0.192	0.05	0.95	0.584	0.35	1.29	**0.015**
Fumaric acid	6.506–6.519(s)	0.14	1.33	0.267	0.12	0.42	0.345	0.15	0.32	**0.019**
Glutamine	2.416–2.494(m)	1.26	0.94	0.319	0.85	1.10	0.110	1.34	1.17	**0.041**
Valine	1.017–1.050(d)	1.59	0.90	0.211	0.17	1.03	0.504	1.86	1.14	**0.041**

*VIP* variable importance in projection, *IgE* immunoglobulin E, *ppm*, parts per million, *s* singlet, *t* triplet, *m* multiplet, *d* doublet.

*VIP score were obtained from PLS-DA.

^†^Fold changes were calculated by dividing the value of metabolites in children with lowly or highly sensitized asthma by controls, and in asthmatic children with by without IgE sensitization.

^‡^All FDR-adjusted *P* values < 0.05, which is in bold, are significant.

### Correlation between blood metabolites and serum IgE levels

**Figure 1:**
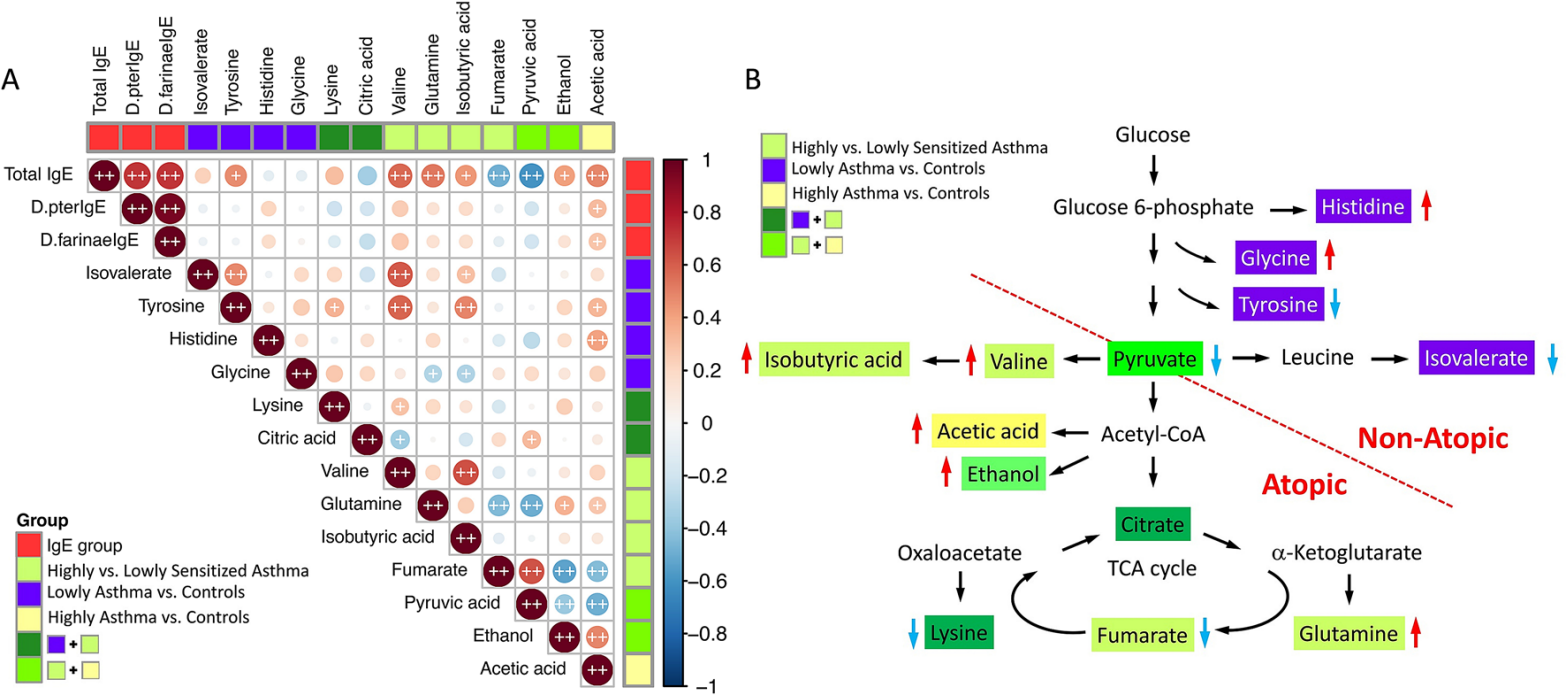
Heatmap of Spearman’s rank correlation coefficients between metabolites associated with lowly and highly sensitized asthma (**A**) and metabolic pathways of metabolites associated with atopic and non-atopic asthma (**B**). Red color represents positive correlations; blue color represents negative correlations; red arrow represents increase; blue arrow represents decrease. + symbol means a *P*-value < 0.05; ++ symbol means a *P*-value < 0.01.

Spearman’s correlation analysis showed significant correlations between metabolites related to highly sensitized asthma and total serum IgE levels (Fig. 1A). Total serum IgE levels were strongly correlated with serum *D. pteronyssinus*- and *D. farinae*-specific IgE levels (*P* < 0.01). Furthermore, there was a strong positive correlation between metabolites associated with highly sensitized atopic asthma (valine, isobutyric acid, and acetic acid) and lowly sensitized non-atopic asthma (isovalerate, tyrosine, and histidine), respectively (*P* < 0.01). Further metabolic pathway analysis revealed amino acid (alanine, aspartate and glutamate) and carbohydrate (glycolysis or gluconeogenesis and butanoate) metabolisms, uniquely in highly sensitized atopic asthma (Table S1). Metabolic pathway analysis showed that highly sensitized asthma associated metabolites were mainly enriched in pyruvate and acetyl-CoA metabolisms (Fig. 1B).

### Identification of airway bacterial community composition and abundance

The reads obtained from the airway microbial sequencing were collected and analyzed. There were no differences in the bacterial richness (Chao1 index) and diversity (Shannon index) regarding the IgE sensitization related to asthma (Fig. S2A). The taxonomic classification of the airway microbiota showed a high prevalence of phylum Firmicutes (42.8% of the total number of sequences obtained) and the most common genera in the airway microbiota were *Streptococcus* (24.3%) of the phylum Firmicutes followed by those of the genus *Prevotella* (9.1%) of the phylum Bacteroidetes, and *Fusobacterium* (5.7%) of the phylum Fusobacteria (Fig. S2B). Clustered heatmap showed the predominantly abundant taxa of airway microbiota and their relevance to lowly and highly sensitized asthma (Fig. S2C). Although there were no statistically significant difference in airway taxa composition between asthma and healthy controls, the abundances of genera *Dialister*, *Streptococcus*, *Prevotella*, *Tannerella*, *Atopobium*, and *Ralstoni*a were predominantly reduced in children with highly sensitized asthma.

### Association between blood metabolome and airway microbiome

**Figure 2:**
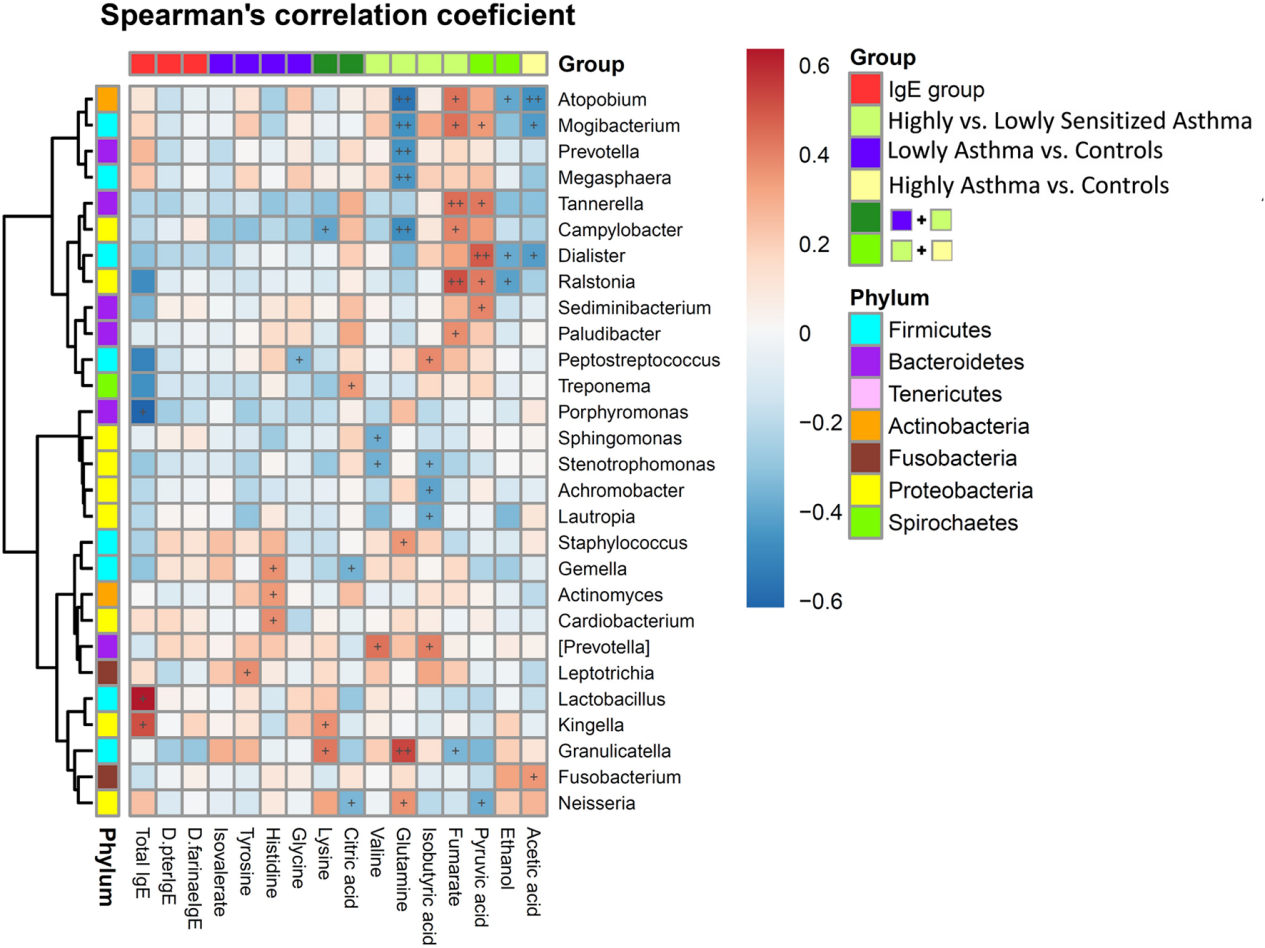
Heatmap of Spearman’s rank correlation coefficients between blood metabolites related to asthma and airway bacterial genera. Red color represents positive correlations; blue color represents negative correlations. + symbol means a *P*-value < 0.05; ++ symbol means a *P*-value < 0.01.

Spearman’s rank correlation coefficients of blood metabolites with bacterial genera grouped by phyla are shown in Fig. 2. The metabolites associated with highly sensitized asthma were strongly correlated with airway microbiota. Acetic acid was negatively correlated with the genus *Atopobium* but positively correlated with the genus *Fusobacterium*. Glutamine was strongly negatively correlated with the genera *Atopobium*, *Mogibacterium*, *Prevotella*, *Megasphaera*, and *Campylobacter*. Fumarate and pyruvic acid were positively correlated with the genus *Ralstonia* and *Dialister* respectively.

## Discussion

Allergic reactions with IgE production are integral to the pathogenesis of atopic diseases. A strong correlation between total serum IgE levels and mite-specific IgE levels indicates that total serum IgE levels can represent the environmental aeroallergen exposure contributing to asthma^8^. In this study, the reduced pyruvic acid in parallel with increased acetic acid levels associated with highly sensitized atopic asthma suggest pyruvate metabolism to energy through acetyl-CoA may play a crucial role in IgE production in response to allergens.

Amino acids contribute to various antioxidant and immunological activities relevant to asthma pathogenesis^9^. Histidine is critical in an enzymatic reaction for histamine which is known for acute allergic reactions in asthma^10^. Glycine is a great supplement to reduce inflammation. *N*-Acetyltyrosine is acetylated from l-tyrosine and is associated with eosinophil activity for asthma^11^. A strong association of these amino acids with lowly sensitized asthma in this study indicates that the glycolysis and amino acid metabolism may play roles in the allergic manifestation without the accompanied increase of serum IgE levels.

Several studies have linked the airway microbiome alterations to atopy and asthma phenotype^12^. Branched short-chain fatty acids (BSCFAs) result from microbial catabolism of the amino acids including valine, isoleucine and leucine^13^. In this study, isobutyric and isovaleric acids, microbial-derived metabolites, were strongly associated with highly and lowly sensitized asthma, respectively. Furthermore, a strong correlation of isovalerate with valine and isobutyric acid indicates that microbial-derived BSCFAs may take part in the molecular immune responses contributing to different endotypes of asthma.

A modulation between airway dysbiosis and responses to HDM plays a role in susceptibility to allergic airway diseases^5^. IgE reactivity to antigens from bacteria is common in patients with airway allergies. HDM has reported to serve as carriers of bacteria and the microbial antigens for the induction of IgE sensitization^14^, which may particularly explain the predominant correlation of airway microbiota with metabolites associated with highly sensitized atopic asthma in this study. Acetic acid, a short-chain fatty acid (SCFA), is produced due to the fermentation process of bacteria in the gut. A strong correlation of acetic acid with mite-specific IgE levels and atopic asthma suggests a potential role of certain acetate-producing bacteria associated with HDM exposure for IgE-mediated childhood asthma.

Acetyl-CoA metabolism via either the acetate or the ethanol branches is governed by Gram-negative anaerobes^15^. *Fusobacterium* is a genus of anaerobic Gram-negative bacteria of the respiratory flora with increased invasive properties in an inflammatory setting^16^. There is also a higher abundance of *Fusobacterium* spp. in asthmatic children^17^. A significant positive correlation between acetic acid and *Fusobacterium* spp. in this study implies the possibility of the host–microbial interactions for the clinical variants of asthma phenotypes.

Glutamine is a metabolite of central importance in bacterial physiology, which favors the growth of the Bacteroidetes family of bacteria^18^. In this study, glutamine was strongly associated with higher IgE levels but with a reduced abundance of the genus *Prevotella* of the phylum Bacteriodetes, which is compatible with previous studies showing that asthmatic children have a lower abundance of *Prevotella*^19^. In addition, *Atopobium* spp. were both strongly correlated with acetic acid and glutamine. *Atopobium* is a member of the oral flora and has been described previously in the obstructive airway diseases^20^. However, the specific presence of *Atopobium* in childhood asthma remains to be elucidated.

A major limitation of this study is its relatively small sample size. Despite this, asthmatic children and healthy controls are physician-diagnosed and confirmed from a birth cohort with short-interval follow-up and long-term prospects for allergies, yielding valid and important results. Another limitation is the general difficulty in bacterial species level identification by using 16S rRNA sequencing. However, an age-matched comparison design in this study eliminates the dissimilarities in the microbial compositions across a wide age range. Furthermore, any contamination in microbiome experiments will not have systematically influenced our results because of using the same protocols and procedures for cases and control subjects. Most importantly, an integrated metabolome and microbiome analysis provides a comprehensive overview of the regulatory network of host–microbial interactions in early childhood asthma.

In conclusion, the metabolic pathway of pyruvate and acetyl-CoA from glucose metabolized to acetic acid is strongly associated with HDM-specific IgE responses in children with asthma. In contrast, the glycolysis and amino acid metabolism play a role in a reaction involving other components of the immune system apart from IgE antibodies for asthma. A strong correlation of metabolites related to highly sensitized atopic asthma with particular subsets of airway microbiota provides evidence for the host–microbial interactions in IgE-mediated childhood asthma. However, further studies with larger sample sizes are required to examine the functional significance of these observations.

## Methods

### Study population

A cross-sectional case–control study was conducted to investigate the blood metabolomic and airway microbial profiles in children aged 3 to 4 years diagnosed with asthma alone and healthy controls from a birth cohort of The Prediction of Allergies in Taiwanese Children (PATCH). Asthma was physician-diagnosed as having the symptoms with shortness of breath, coughing, and recurrent wheezing, based on the guidelines of the Global Initiative for Asthma^5^. However, episodes of wheezing induced by viral infections of the upper respiratory tract, few or no interval wheeze symptoms between viral illnesses, were excluded from asthma^21,22^. Non-atopic asthma was defined as total serum IgE levels < 100 kU/L, whereas atopic asthma was defined as total serum IgE levels ≥ 100 kU/L with highest positive predictive value for atopy and negative predictive values for asthma^23^. Children with lowly sensitized asthma was defined as the specific IgE levels against *D. pteronyssinus* less than 3.5 kU/L (class 2), whereas highly sensitized asthma was defined as *D. pteronyssinus*-specific IgE levels ≥ 3.5 kU/L (class 3–6) to confirm the presence of allergy^24^. Healthy children without a history of atopic conditions with total serum IgE levels < 100 kU/L and with *D. pteronyssinus* less than 0.70 kU/L (class 1) were enrolled as controls. Information regarding demographic data, family atopy history, passive smoking, and household income related to asthma were collected. This study was approved by the Ethics Committee of Chang Gung Memory Hospital (No. 201701959B0C502). All experiments in this study were performed in accordance with the relevant guidelines and regulations and written informed consent was obtained from the parents or guardians of all study subjects.

### Sample collection and preparation

Blood samples were collected at the outpatient clinics and throat swabs were collected using sterile cotton swabs rubbed at least three times around the oropharynx with swab rotation without interruption. Throat swab sampling was performed in subjects without receiving probiotics or antibiotics therapy for at least 4 weeks prior to the sampling. These samples were frozen immediately and stored at − 80 °C until required.

### Measurement of serum and allergen-specific IgE levels

Total serum and allergen-specific serum IgE levels were examined as described in our previous study^25^. Total serum IgE level was measured by ImmunoCAP (Phadia, Uppsala, Sweden), whereas specific IgE levels to *Dermatophagoides pteronyssinus* and *Dermatophagoides farinae* allergens were determined using a commercial assay for IgE (ImmunoCAP Phadiatop Infant; Phadia)^26^ and recorded as a continuous variable.

### ^1^H–NMR spectroscopy

Plasma samples prior to spectrum acquisition were prepared as described previously^27^. A 500 μL of plasma was mixed with 500 μL phosphate buffer in deuterium water containing 0.08% 3-(trimethylsilyl)-propionic-2,2,3,3-d_4_ acid sodium salt (TSP) as an internal chemical shift reference standard. An aliquot of 600 μL was transferred to a standard 5 mm NMR tube for further analysis. ^1^H-NMR spectra were acquired using a Bruker Avance 600 MHz spectrometer (Bruker-Biospin GmbH, Karlsruhe, Germany) at Chang Gung Healthy Aging Research Center, Taiwan.

### NMR data processing and analysis

The raw ^1^H-NMR spectra were spectra processed, ppm calibrated, baseline corrected, aligned, spectra bucketed and data normalized using NMRProcFlow online software^28^. Spectra bucketing was performed using intelligent bucketing and variable size bucketing^29^. Chenomx NMR Suite 8.0 professional software (Chenomx Inc., Edmonton AB, Canada) was then used to identify metabolites. As previously established NMR data analysis methods^27^, the ^1^H-NMR spectra data were generalized log transformed (glog) and pareto-scaled, and metabolites used for discrimination between the groups were identified using partial least squares-discriminant analysis (PLS-DA) in MetaboAnalyst (version 4.0). Metabolites with a variable importance in projection (VIP) score ≥ 1.0 or a *P*-value < 0.05 were selected. Functional metabolite pathways were analyzed based on the Kyoto Encyclopedia of Genes and Genomes (KEGG) database^30–32^.

### 16S rRNA gene sequencing and microbiome data analysis

Throat swab bacterial DNA was extracted using a FastDNA Spin Kit for Soil (MP Biomedical, Solon, OH, USA) following the manufacturer’s instructions. The variable region V3-V4 encoded for 16S rRNA gene was amplified by using primer 341F/805R with the barcodes^5^. Amplicon sequencing was performed on the Illumina HiSeq 2500 platform (Illumina, Inc., San Diego, CA, USA) and the entire target was assembled with 250 bp paired-end reads by using FLASH^33^. Analysis of microbiome data was performed using the software ‘‘Quantitative Insights into Microbial Ecology’’ (QIIME 1.9.1)^34^. Assembled sequences were clustered into operational taxonomic units (OTUs) using UPRASE software at 97% sequence identity, and taxonomy classification was assigned based on the latest Greengenes database^35^. The species richness of each sample was calculated with the Chao1 index and the species diversity was evaluated with Shannon index^5^. The heatmap of OTU relative abundance was generated to visualize the distribution of bacterial communities among non-IgE asthma, IgE asthma, and healthy controls and their abundance differences were tested between groups using the MetaStat method^36^.

### Statistical analysis

Comparisons of baseline characteristics between asthmatic patients and healthy controls were performed with univariable parametric and non-parametric tests as appropriate. Differences in metabolites were assessed with the MetaboAnalyst web server using Mann–Whitney test. A false discovery rate (FDR) of 5% was applied to correct for multiple tests. The correlation coefficients between with blood metabolites and between with airway bacterial compositions and serum IgE levels were calculated using Spearman’s correlation test in R software (Lucent Technologies, NJ, USA, version 3.3.1). Statistical analysis was performed by using the Statistical Package for the Social Sciences (SPSS Statistics for Windows Version 20.0; Armonk, NY). All statistical hypothesis tests were 2-tailed and a *P*-value < 0.05 was considered significant.

## Supplementary Information

Supplementary Information.

## Data Availability

The datasets generated during and/or analyzed during the current study are not publicly available duo to the personal privacy of subjects but are available from the corresponding author on reasonable request.
