# Supplementary material for: Integrated metabolic and microbial analysis reveals host–microbial interactions in IgE-mediated childhood asthma
Source: Sci Rep. 2021 Dec 3;11:23407. doi: 10.1038/s41598-021-02925-5 (PMC8642522; doi:10.1038/s41598-021-02925-5)
Supplement: Supplementary file 1 — Supplementary Information. [file 41598_2021_2925_MOESM1_ESM.docx]

**Supplementary information**

**Integrated metabolic and microbial analysis reveals host-microbial interactions in IgE-mediated childhood asthma**

Chih-Yung Chiu, Mei-Ling Cheng, Meng-Han Chiang, Chia-Jung Wang, Ming-Han Tsai & Gigin Lin


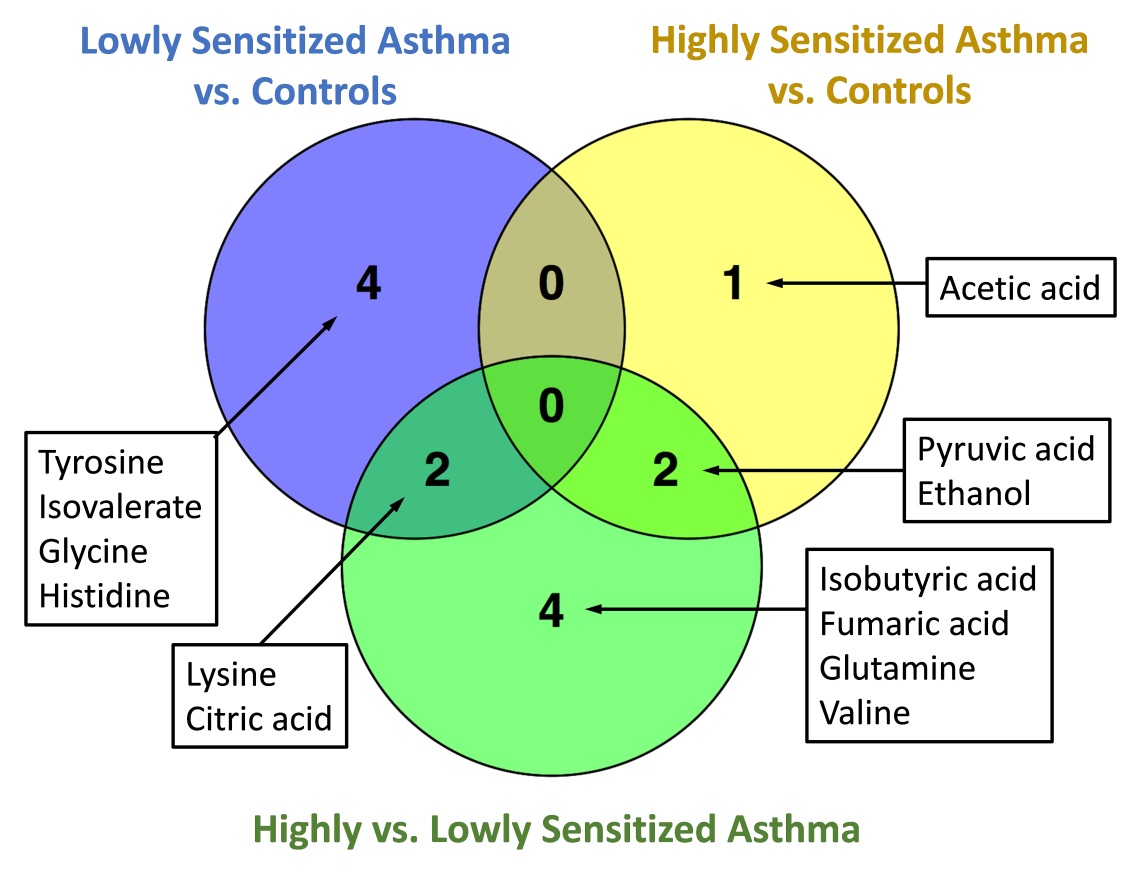


**Supplementary Figure S1.** Venn diagram of metabolites associated with lowly and highly sensitized asthma. The total number and names of metabolites in each set and the overlapping areas are indicated.

**Supplementary Table S1.** Metabolic pathway and function analysis of highly sensitized atopic and lowly sensitized non-atopic asthma.

| Pathway Name | Metabolites | Total | Hits | Raw *P* | Holm *P* | Function |
| --- | --- | --- | --- | --- | --- | --- |
| **Highly sensitized asthma** |  |  |  |  |  |  |
| Alanine, aspartate and glutamate metabolism | Pyruvic acid, glutamine, fumaric acid | 24 | 3 | 2.97E-05 | 0.002 | Amino acid metabolism |
| Glycolysis or Gluconeogenesis | Pyruvic acid, ethanol, acetic acid | 31 | 3 | 6.54E-05 | 0.005 | Carbohydrate metabolism |
| Butanoate metabolism | Pyruvic acid, butyric acid, fumaric acid | 40 | 3 | 1.42E-04 | 0.011 | Carbohydrate metabolism |
| **Lowly sensitized asthma** |  |  |  |  |  |  |
| Aminoacyl-tRNA biosynthesis | Histidine, glycine, lysine | 75 | 3 | 2.78E-05 | 0.022 | Genetic Information Processing; Translation |

Total is the total number of compounds in the pathway; the Hits is the actually matched number from the user uploaded data; the Raw *P* is the original *P* value calculated from the enrichment analysis; the Holm *P* is the *P* value adjusted by Holm-Bonferroni method.

**
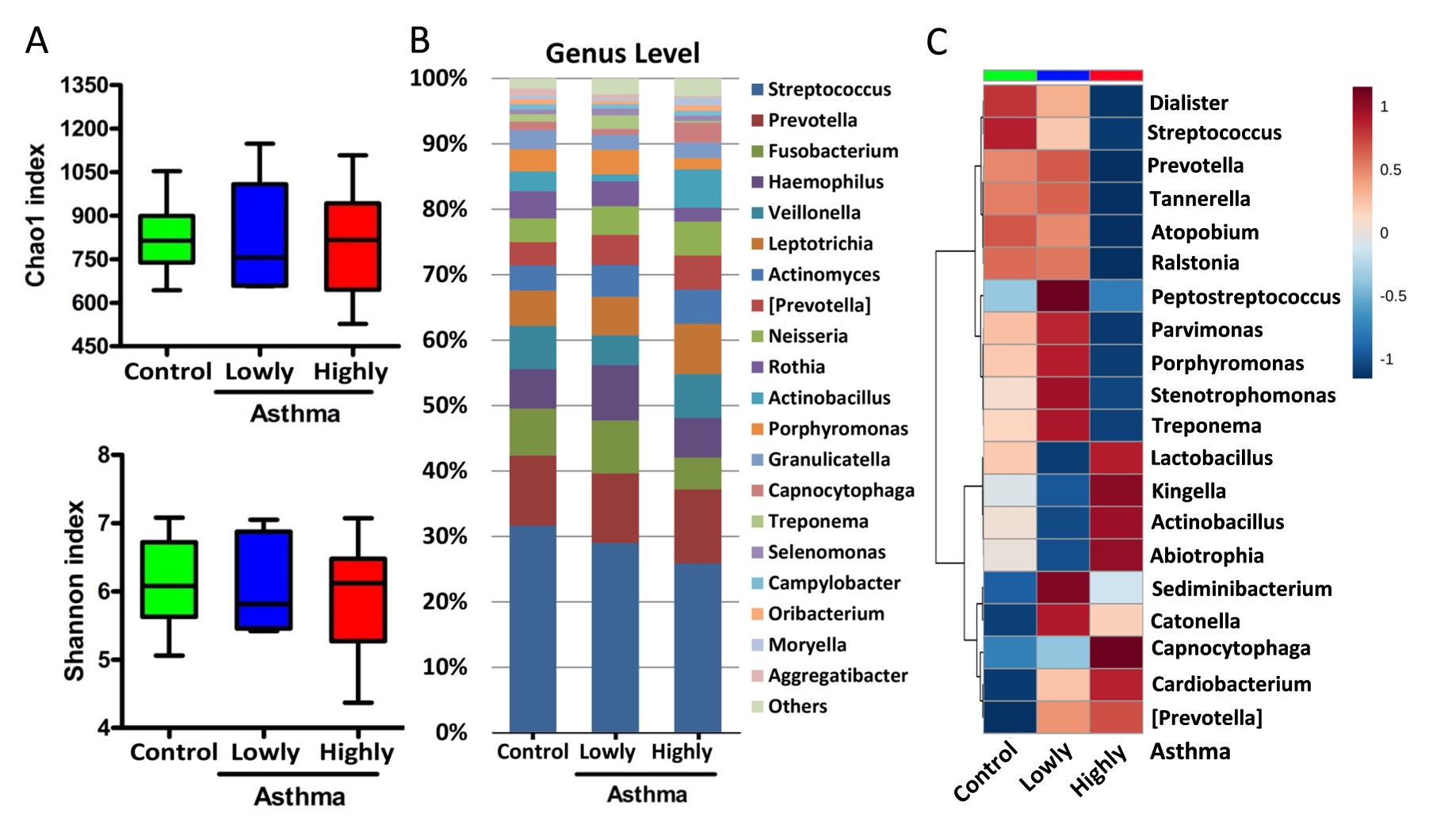
**

**Supplementary Figure S2.** Estimates of bacterial richness (Chao1 index) and diversity (Shannon index) (A), bacterial composition and abundance at genus levels (B), and heatmap of predominantly abundant taxa of airway microbiota (C). Each row represents the distribution of the top 20 abundant genera in children with lowly and highly sensitized asthma and healthy controls. The color of each cell indicates the relative abundance of bacterial genera.
